# The Role of Mitochondria-Targeting miRNAs in Intracerebral Hemorrhage

**DOI:** 10.2174/1570159X20666220507021445

**Published:** 2023-04-12

**Authors:** Ilgiz Gareev, Ozal Beylerli, Yanchao Liang, Enzhou Lu, Tatiana Ilyasova, Albert Sufianov, Galina Sufianova, Huaizhang Shi, Aamir Ahmad, Guang Yang

**Affiliations:** 1 Federal Centre of Neurosurgery, Tyumen, Russia;; 2 Department of Neurosurgery, The First Affiliated Hospital of Harbin Medical University, Harbin, 150001, China;; 3 Institute of Brain Science, Harbin Medical University, Harbin, 150001, China;; 4 Bashkir State Medical University, Ufa, Republic of Bashkortostan, 450008, Russia;; 5 Department of Neurosurgery, Sechenov First Moscow State Medical University (Sechenov University), Moscow, Russia;; 6 Department of Pharmacology, Tyumen State Medical University, Tyumen, Russia;; 7 Interim Translational Research Institute, Academic Health System, Hamad Medical Corporation, Doha, Qatar;; 8 Рeoples’ Friendship University of Russia (RUDN University), 6 Miklukho-Maklaya Street, Moscow, 117198, Russian Federation

**Keywords:** Intracerebral hemorrhage, miRNA, therapeutic target, biomarker, pathogenesis, mitochondria, mitochondrial dysfunction

## Abstract

Non-traumatic intracerebral hemorrhage (ICH) is the most common type of hemorrhagic stroke, most often occurring between the ages of 45 and 60. Arterial hypertension (AH) is most often the cause of ICH, followed by atherosclerosis, blood diseases, inflammatory changes in cerebral vessels, intoxication and vitamin deficiencies. Cerebral hemorrhage can occur by diapedesis or as a result of a ruptured vessel. AH is difficult to treat, requires surgery and can lead to disability or death. One of the important directions in the study of the pathogenesis of ICH is mitochondrial dysfunction and its regulation. The key role of mitochondrial dysfunction in AH and atherosclerosis, as well as in the development of brain damage after hemorrhage, has been acknowledged. MicroRNAs (miRNAs) are a class of non-coding RNAs (about 18-22 nucleotides) that regulate a variety of biological processes including cell differentiation, proliferation, apoptosis, *etc.*, primarily through gene repression. There is growing evidence to support dysregulated miRNAs in various cardiovascular diseases, including ICH. Further, the realization of miRNAs within mitochondrial compartment has challenged the traditional knowledge of signaling pathways involved in the regulatory network of cardiovascular diseases. However, the role of miRNAs in mitochondrial dysfunction for ICH is still under-appreciated, with comparatively much lesser studies and investigations reported, than those in other cardiovascular diseases. In this review, we summarize the up-to-date findings on the published role miRNAs in mitochondrial function for ICH, and the potential use of miRNAs in clinical settings, such as potential therapeutic targets and non-invasive diagnostic/prognostic biomarker tools.

## 1 INTRODUCTION

Non-traumatic intracerebral hemorrhage (ICH) is local bleeding into the parenchyma of the brain, which occurs as a result of a rupture of cerebral blood vessels. ICH usually develops when an atherosclerotic cerebral artery ruptures, the wall of which has undergone changes as a result of a prolonged existing increase in blood pressure [1, 2]. The problem of early diagnosis, prognosis and treatment of ICH is one of the most important in modern medicine. Despite the vast experience of modern neurosurgery and neurology in the treatment of patients with ICH, the tactics of managing patients are still controversial, and indications for various methods of therapy need to be clarified. Until now, neither conservative nor surgical treatment methods have a clear advantage. Therefore, the study of the molecular mechanisms of the pathogenesis of ICH will deepen the understanding of the course of ICH and clarify some issues of diagnosis and treatment tactics.

The mechanisms of ICH development include impaired blood-brain barrier (BBB) function and cerebral edema, cell apoptosis, inflammation, oxidative stress, activation of signaling pathways that regulate angiogenesis, and suppression of signaling pathways responsible for maintaining the phenotype of vascular smooth muscle cells (VSMCs) [3, 4]. Mitochondria are known to play an important role in the pathogenesis damage during ICH through the generation of reactive oxygen species (ROS), mitochondrial dysfunction, and the mitochondrial pathway of apoptosis [5]. The involvement of microRNAs (miRNAs) has been demonstrated for each of the listed mechanisms, highlighting the significant contribution of miRNAs to the development of ICH [6-8]. MiRNAs are short, on average 18-22 nucleotides, single-stranded non-coding RNAs that post-transcriptional regulate gene expression by binding to the 3'-untranslated region (3'-UTR) of the messenger RNA (mRNA) target, which ultimately leads to decreasing protein expression by blocking translation and/or promoting degradation of the target mRNA [9]. In addition, in recent years, the attention of researchers has been attracted by the study of changes in the expression levels of circulating miRNAs in various biological fluids of the human body for their potential consideration as non-invasive diagnostic and prognostic markers of cardiovascular diseases, including stroke [10]. In this review, we highlight the up-to-date findings on the theoretical role miRNAs in mitochondrial function for ICH, their targets, and mechanisms of action, and summarizes the latest advances in the context of the potential application of miRNAs in clinical practice, particularly, the possibility of their use for early diagnosis, prognosis, and therapy for ICH.

## 2 MIRNAS AND MITOCHONDRIA

**Fig. (1):**
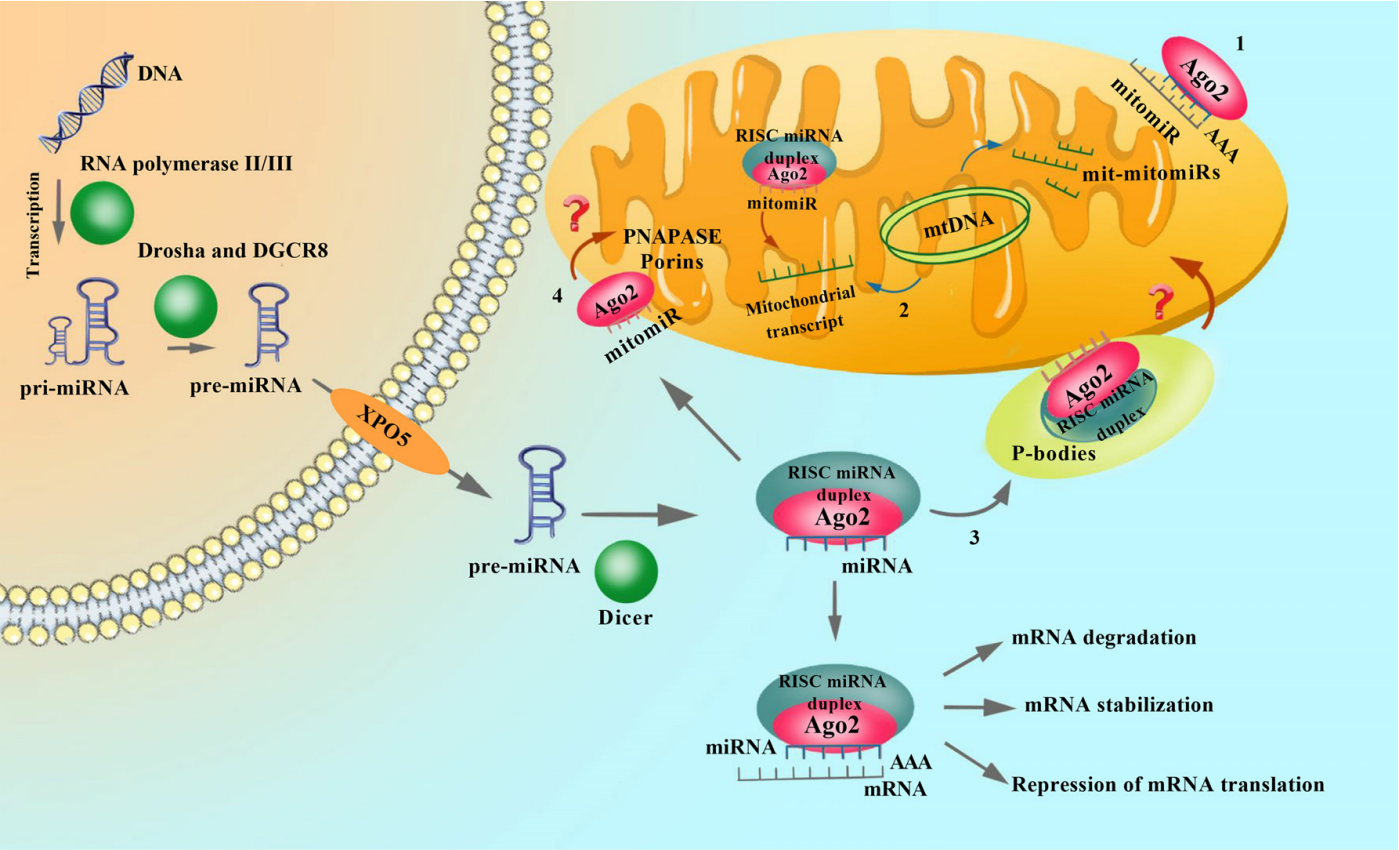
Cellular release of circulating miRNAs into biological fluids (*e.g.* bloodstream). In the cytoplasm, pre-miRNAs as well as mature miRNAs can be incorporated into microparticles, such as extracellular vesicles (EVs) (exosomes and microvesicles) or apoptotic bodies, and can be released from cells. In addition to being incorporated into the EVs, miRNAs are also present in the microparticle-free compartment. These miRNAs are associated with high-density lipoprotein particles (HDLPs) or RNA-binding proteins (miRNA-Argonaute 2 (Ago2) complex). In physiological conditions or diseases, miRNAs can be released passively (during apoptosis or necrosis) or actively, by secretion in the EVs, or miRNA-Ago2 complex through interaction with specific membrane channels or proteins.

MiRNA biogenesis includes several stages (Fig. **1**). At the first stage, miRNA is transcribed by RNA polymerase II and III where is this enzyme synthesizes a long double-stranded primary miRNA (pri-miRNA) from independent genomic transcriptional units or from introns of protein-coding genes. In the second stage, Drosha (Class 2 ribonuclease III enzyme) excises the shorter precursor miRNA (pre-miRNA) from the pre-miRNA, which is 60 nucleotides long. One or more pre-miRNAs can be formed from one pri-miRNA. The Drosha is a component of a large microprocessor complex. This nuclear complex exists in two forms: a 600 kDa structure, the function of which has not yet been established, and a heterodimer that includes Drosha and the RNA-binding protein DGCR8. The subcellular distribution of miRNA-synthesizing enzymes and their substrates shows that pre-miRNAs are formed in the nucleus, while pre-miRNAs are in the cytoplasm. Transport of pre-miRNA from the nucleus to the cytoplasm through nuclear pores occurs with the participation of the nuclear transport receptor Exportin 5 (XPO5). In addition to the transport function, the XPO5 protects pre-miRNAs from degradation by exonucleases. It should be noted that the biogenesis of mature miRNAs can also occur with the participation of nucleases from other signaling pathways, including the main mechanism of RNA degradation and pre-miRNA splicing factors. MiRNAs carry out post-transcriptional gene silencing [3, 11, 12]. They are able to regulate the intensity of the processes of transcription, RNA processing, and translation through complementary interactions with DNA or mRNA in various cells and tissues [13]. Each miRNA has binding sites for many mRNAs, and one mRNA is a possible target for several miRNAs. Thus, miRNAs and mRNAs form a complex network of regulatory interactions that are involved in the epigenetic modification of gene expression [11-13].

The main function of mitochondria is the synthesis of adenosine triphosphate (ATP), a universal form of energy in any living cell. Oxidative phosphorylation occurs in the inner membrane of mitochondria and consists of 4 stages: 1) conversion of pyruvate and fatty acids from the cytoplasm into mitochondria into acetyl-CoA; 2) oxidation of acetyl-CoA in the Krebs cycle, leading to the formation of nicotinamide adenine dinucleotide (NADH); 3) transfer of electrons from NADH to oxygen along the respiratory chain; 4) the formation of ATP as a result of the activity of the membrane ATP-synthetase complex [14, 15]. In addition to ATP synthesis, oxidative phosphorylation is an endogenous source of ROS: superoxide, hydrogen peroxide, and hydroxyl radical [14, 15]. Prolonged exposure to ROS on the cell leads to oxidative damage to proteins, lipids and nucleic acids, and acute exposure to inactivation of Fe-S centers of enzymatic complexes of oxidative phosphorylation and the enzyme of the tricarboxylic acid cycle - aconitase, which leads to a decrease in ATP production [16]. Exposure to ROS leads to the accumulation of multiple mutations, a decrease in the rate of oxidative phosphorylation, and an even greater accumulation of ROS. All this ultimately disrupts the functioning of the cell, causing programmed cell death – apoptosis [17]. Mitochondria play an important role in ensuring the normal functioning of endothelial cells (ECs), when mitochondria not only produce ATP, but also regulate the work of cellular messengers such as calcium and ROS [18].

It is known that miRNAs regulate the metabolism and biogenesis of mitochondria both under physiological conditions and under pathology. In mitochondria, there are two types of miRNAs that control the mitochondrial genome: 1) nuclear miRNAs, which are transported to mitochondria, and 2) mitochondrial miRNAs (mitomiRNAs), which inhibit transfer RNA (tRNA) functions (Fig. **1**) [19, 20].

It is known that systematic profiling analysis performed on samples of nuclear and cytoplasmic RNA fractionation concluded that the majority of miRNAs are present in the nucleus [21]. These show that most if not all miRNAs have the ability to move between the nucleus and the cytoplasm. This finding is supported by the nuclear localization of Argonaute (AGO) proteins and the recent discovery that trinucleotide repeat-containing gene 6A protein (TNRC6A is a component of the RNA-induced silencing complex (RISC)) is a nuclear and cytoplasmic shuttle protein that facilitates Ago transport into the nucleus [22]. The role of nuclear miRNAs has been relatively neglected. Indeed, most studies have focused on the action of miRNAs on post-transcriptional gene regulation. However, it is important to recognize that miRNAs move from the cytoplasm to the nucleus. Even if repression of specific nuclear miRNAs is achieved, the observed functional loss may be due to a general depletion of cytoplasmic miRNAs that are inhibited when they enter the nucleus. Recent studies have revealed that nuclear miRNAs can function in an unconventional manner to regulate the biogenesis and functions of non-coding RNAs, adding a new layer of complexity to our understanding of gene regulation [23, 24].

The discovery of miRNAs localized in mitochondria, called mitomiRNAs, raised the question of the mechanisms of their translocation from the cytoplasm and the possibility of their formation directly with the participation of mitochondrial DNA (mDNA) [25]. These processes require energy supply and are ATP-dependent. It was found that ATP production during oxidative phosphorylation in mitochondria is regulated with the participation of miR-156, miR-16, miR-195, and miR-424 [26]. In addition, miR-181c, miR-210, and miR-338 regulate individual links in the electron transport chain in mitochondria [27]. However, it is only very recently that light has begun to be shed on how miRNAs are involved in the regulation of mitochondrial function. In fact, in addition to the presence of miRNAs in the cytosol, which regulate mRNA-targets that encode proteins involved in mitochondrial-related activities, it has recently been reported that a small number of miRNAs are present in the mitochondrial matrix itself. Thus, miRNA-mediated mitochondrial dysfunction is responsible not only for the onset, but also for the progression of various human diseases, including cardiovascular diseases (Table **1**) [28-35].

## 3 MIRNA AND MITOCHONDRIAL PERMEABILITY TRANSITION PORE

Mitochondrial permeability transition pore (mPTP) is a multiprotein complex consisting of cyclophilin D (CyPD), mitochondrial peptide proline trans isomerase, voltage-dependent anion channel 1(VDAC1), adenine nucleotide transporter (ANT), and is a non-selective channel that plays a significant role in calcium exchange between mitochondria and the environment [36]. The opening of the mPTP channel is induced by calcium ions (Ca^2+^) of the mitochondrial matrix. It is believed that the entry and exit of Ca^2+^ from mitochondria occur in different ways [37]. Calcium plays a regulatory role in the functioning of the pore - it activates its opening from the side of the matrix, but, on the contrary, blocks it from the outside of the mitochondrial membrane. Regular opening of mPTP plays a key physiological role in maintaining a healthy internal mitochondrial environment [38]. The mitochondrial pore functions by changing the conformation of its constituent proteins, thereby regulating the activity of metabolic processes. The opening of the mitochondrial pore occurs in certain pathological conditions, such as strokes, traumatic brain injury (TBI), neurodegenerative diseases, *etc.* [39-41]. At high ROS levels, the constant opening of mPTP will trigger the release of ROS, resulting in mitochondrial dysfunction [35, 36]. The mPTP is likely to play a critical role in these processes, as increased ROS activates mPTP opening, which further increases ROS production. This positive feedback mechanism ultimately leads to excess accumulation of ROS. The accumulation of ROS, in turn, damages nuclear DNA, activates pro-apoptotic signaling pathways, and stimulates cellular senescence [42, 43]. However, in some cases, ROS can activate protective pathways, reduce the load on mitochondria, and increase cell lifespan [44, 45]. It is currently believed that mPTP plays an important role in integrating the effects of ROS and therefore may play a vital role in endothelial dysfunction. In addition, the transfer of ROS from damaged mitochondria to neighboring mitochondria results in uncontrolled damage. mPTP activation may also be a potential mechanism for necrosis and apoptosis [38]. The fate of a cell, for example, after an ICH, depends on the extent and duration of mPTP opening [41]. In general, mitochondria are the main sites for ROS production and are involved in ROS amplification.

Differential expression and function of miRNAs have been linked to the mitochondrial dysfunction, including opening of mPTP, of several diseases [39-41]. However, how miRNAs contribute to the opening of mPTP in ICH remains elusive. Chaudhuri *et al.* demonstrated that miR-7 prevents depolarization of mitochondria in response to 1-methyl-4-phenylpyridinium (MPP+) by directly downregulating VDAC1 of Parkinson’s disease (PD) *in vivo* and *in vitro* [46]. Consequently, MPP+ - induced calcium efflux and cytochrome c release from mitochondria to cytosol were attenuated by miR-7. The authors showed that miR-7 regulates the function of mPTP by targeting VDAC1. Consequently, miR-7 prevents MPP+-induced opening of mPTP in PD model, thereby conferring neuroprotection. Previous studies on ICH found that the increase in miR-7 expression in ICH causes the activation of the various signal pathways (phosphoinositide 3-kinase (PI3K)/AKT, epidermal growth factor receptor (EGFR)/ signal transducer and activator of transcription 3 (STAT3), and toll-like receptor 4 (TLR4)), resulting in protection against brain injury after hemorrhage [47-49]. In another study, Fu *et al.* showed that inhibition of miR-224 expression may suppress neuronal apoptosis *via* targeting 3’-UTR mRNA spastic paraplegia type 7 (SPG7) and thereby preventing mPTP formation in ischemic stroke (IS) [50]. SPG7 is one of the targets of miR-224, and it is required for mPTP formation in several cell types, which promotes Ca^2+^ - induced mPTP opening. This study provides a new perspective in understanding the pathogenesis of IS, with regard to more detailed molecular mechanism and cross with miR-224/SPG7 signaling pathway of mPTP opening in ICH that still needs to be further explored. This study provides a new perspective on understanding the pathogenesis of the cerebrovascular disease, with regard to a more detailed molecular mechanism *via* miR-224/SPG7 signaling pathway of mPTP opening in ICH that needs to be explored.

Further research is needed to delineate the mechanisms of miRNAs involved in mPTP, and to establish a better understanding of ICH.

## 4 MICRORNAS INVOLVED IN CALCIUM REGULATION

The ability of mitochondria to act as a Ca^2+^ buffer has important implications for the nature of cellular signals [51]. In addition, mitochondria are also the main repository of cellular Ca^2+^, and Ca^2+^ homeostasis is basic for a wide range of cellular functions, such as control of oxidative phosphorylation (OXPHOS), modulation of cytosolic Ca^2+^ signals (cytoCa^2+^), cell death, secretion and the production of ROS [51-53]. As mentioned above, mitochondrial Ca^2+^ (mCa^2+^) also stimulates cell death pathways, such as apoptosis and necrosis, and mCa^2+^ overload or mitochondrial depolarization can open the mPTP [34, 52]. Disruption of mCa^2+^ cycling is implicated in numerous acquired diseases such as IS and ICH, heart failure, neurodegenerative disease, diabetes mellitus, and tumors [53]. Understanding the mechanisms responsible for mCa^2+^ exchange therefore holds great promise for the treatment of these diseases such as ICH. ICH induces primary and secondary damage to the brain. Blood extravasation during ICH damages neurons, glial cells, and blood vessels cells [2, 3]. In fact, ICH is characterized by abnormal mCa^2+^ handling and poor energy production, which ultimately leads to the dysfunction of all these cells and their death [54]. This increased mCa^2+^ concentration was related to mitochondrial dysfunction seen as membrane depolarization, reduced ATP production, and ROS generation. In IS and ICH (secondary damage), both neurons and glial cells are characterized by increased mCa^2+^ uptake resulting in excessive ROS production and mPTP opening, which ultimately leads to cell death [34, 55, 56].

Recent studies suggest that nuclear miRNAs are able to translocate into the mitochondrial compartment and modulate mCa^2+^ uptake [57]. In addition to this subset of miRNAs, there are several miRNAs that are reported to act on target genes that play a role in maintaining mCa^2+^ levels in the cytoplasm [58, 59]. It is known that sodium-calcium exchanger-1 (NCX1) is involved in IS and ICH characterized by a dysregulation of ionic homeostasis [60]. Vinciguerra *et al.* demonstrated miR-103/107 family by reducing NCX1 expression and worsened ischemic damage induced by middle cerebral artery occlusion (MCAO) [61]. More interestingly, using anti-miR-103-1 induced upregulation of NCX1 protein expression, thus exerting a remarkable neuroprotective effect in IS. At the molecular level, the authors demonstrated that in cortical neurons exposed to oxygen and glucose deprivation, the antiporter NCX1, working in the reverse mode, promotes Ca^2+^-influx and favors Ca^2+^-refilling into the endoplasmic reticulum, thus allowing neurons to delay the apoptotic process. This result may provide a novel understanding of how the miR-103/107 family by regulating NCX1 expression contributes to secondary brain injury and recovery from ICH.

The mitochondrial calcium uniporter (MCU) is located in the inner mitochondrial membrane and facilitates the entry of Ca^2+^ into mitochondria from the cytosol. Consequently, MCU plays a prominent role in mitochondrial homeostasis and is a central role in the ICH pathogenesis of the brain [62]. The MCU maintains mitochondrial Ca^2+^ homeostasis under physiological conditions, which is necessary for the survival and energy supply of cells [63]. In recent years, there has been a growing interest in the role of glycemic variability (GV) in stroke outcomes in several studies [64, 65]. Post-stroke hyperglycemia is common in the acute phase of stroke and is considered an independent predictor of adverse clinical outcomes in both IS and ICH [64, 65]. Based on this, Wang *et al.* examined the effect of miR-129-3p on apoptosis of hippocampal neuronal cells *via* the targeting MCU during GV and elucidated the underlying molecular mechanisms involved [66]. Their study provides evidence for an anti-apoptotic effect of miR-129-3p by targeting MCU expression, relieving mitochondrial Ca^2+^ overload and oxidative stress, and inactivating the mitochondrial-dependent intrinsic apoptosis pathway in hippocampal neuronal cells exposed to GV. Moreover, Wang *et al.* showed that miR-129-3p reduced MMP-2 expression in GV-treated hippocampal neuronal cells. It is known that MMPs are zinc- and calcium-dependent endopeptidases that degrade the extracellular matrix (ECM) [67]. Wherein, MMP-2 may play a role in ICH pathology [67]. These results strongly suggest that miR-129-3p protects GV-treated hippocampal neuronal cells by reducing MCU expression and subsequently easing mitochondrial Ca^2+^ overload, ROS synthesis, and mitochondrial-dependent intrinsic apoptosis.

## 5 REGULATE BLOOD–BRAIN BARRIER PERMEABILITY AND MITOCHONDRIAL FUNCTION

BBB is an active interaction between the bloodstream and the CNS. The presence of BBB, on the one hand, limits the transport of potentially toxic and hazardous substances from the blood to the brain; on the other hand, it ensures the transport of gases and nutrients to the brain and the removal of metabolites. The study of the mechanisms of the BBB functioning is one of the key tasks, the solution of which has not only fundamental but also applied significance. Disruption of BBB tight junctions has been well documented in ICH and is considered to be a pathological condition of this disease and plays a key role in disease progression as well [68]. A recent study demonstrates that the mitochondrial mechanisms regulate BBB integrity and permeability *in vitro* and *in vivo* model of ICH [69]. Additionally, several miRNAs have recently been found to regulate BBB integrity and permeability [70, 71]. However, little is known regarding the role of miRNAs in mitochondrial function and BBB permeability. For instance, Bukeirat *et al.* demonstrated that the overexpression of miR-34a breaks down the BBB through inhibition of mitochondrial function *in vitro*. In particular, the overexpression of miR-34a results in an increased BBB permeability and the disruption of tight junctions zonula occludens 1 (ZO-1) in cerebrovascular endothelial cells (CECs) line [72]. Overexpression of miR-34a was impaired mitochondrial oxidative phosphorylation and reduced ATP production in CECs. In addition, bioinformatics analysis revealed a series of potential miR-34a-targeting candidates related to mitochondrial function. Bukeirat *et al.* elucidated that cytochrome c (CYCS) is a miR-34a target, and the overexpression of miR-34a inhibited the CYCS expression and increased the expression of several mitochondria-associated gene candidates, including succinate dehydrogenase subunit c (SDHC), cytochrome B reductase 1 (CYBRD1), cytochrome B5 reductase 3 (CYBRD5), pyruvate dehydrogenase kinase isozyme 1 and 2 (PDK1 and PDK2). This result provides the first description of miRNA affecting mitochondrial activity in BBB integrity and permeability, which could lead to a revision of current miRNAs targets and may lead to the discovery of new mechanisms.

Although interventions and clinical management of stroke have improved, the poor prognosis due to secondary brain injury has not changed in patients with ICH. Secondary damage is manifested both in the structures of the brain tissue and in the cerebral vessels. One of the factors that can form damaging and protective cascades in intracerebral hemorrhagic stroke is the presence of a hematoma that undergoes lysis [2, 3]. It is assumed that substances released during lysis of red blood cells cause delayed edema during hemorrhage, which aggravates the damage. Thus, in the ICH model during the infusion of precipitated erythrocytes, a delayed formation of edema and an increase in BBB permeability (on the 3rd day after hemorrhage) were found, while lysis erythrocytes caused significant edema within a day and also contributed to an increase in BBB permeability [73]. The role of hemoglobin and, especially, oxyhemoglobin (oxyHb) in the development of impaired BBB function and edema is shown in *in vitro* and *in vivo* models of ICH [74]. In a recent study, Li *et al.* used oxyHb-treated SH-SY5Y cells to imitate ICH models *in vitro* [75]. In this study was be transfected miR-137 to exosomes derived from endothelial progenitor cells (EPC-EXs). In addition, using bioinformatic methods was confirmed that cyclooxygenase-2 (COX-2) and prostaglandin E2 (PGE2) was potential target of miR-137. These results indicate that the activation of the COX2/PGE2 pathway reversed the protective effects of exosomal miR-137 against apoptosis and mitochondrial dysfunction in oxyHb-treated SH-SY5Y cells.

## 6 SECONDARY ISCHEMIC INJURY AFTER ICH

Following arterial rupture and parenchymal cerebral hemorrhage, a combination of local compression, cytotoxic injury, cell death, inflammation, and surrounding edema occurs. Imaging studies in patients with ICH have demonstrated these effects at various stages of hemorrhage and in and around the hematoma bed. Despite limited diffusion within the hematoma during the first 2 weeks due to the effect of increased viscosity and the effects of suspicion from blood breakdown products, much more attention was paid to the possibility of ischemia in the surrounding tissues [76, 77]. In microglia, the role of mitochondrial function in neuronal survival can be considered direct. Therefore, mitochondrial function and microglial activation serve reciprocal roles [78].

The brain-enriched miR-181family contains four members as miR-181a, miR-181b, miR-181c, and miR-181d, which play a role in mitochondrial function, redox state, and inflammatory pathways [79, 80]. Overexpression of miR-181a in astrocytes increased the disruption of the mitochondrial membrane potential (ΔΨm) and increased the formation of ROS and the death of neurons as a result of glucose deprivation [81]. Xu *et al.* demonstrated the effect of intracerebroventricular infusion and intravenous injection of anti-181a, a chemically modified inhibitor of 181a, in the treatment of ischemic injury *in vivo* [82]. Anti-miR-181a was effective in abolishing endogenous miR-181a expression and demonstrated a significant neuroprotective effect against ischemic brain injury. This protective effect, including the restoration of motor function and coordination, was maintained during the 28 days of observation of the animals, which is consistent with a decrease in the expression of B-cell lymphoma 2 (Bcl-2) and X-linked inhibitor of apoptosis protein (XIAP) (The Bcl-2 family and XIAP are known to play an important role in the evolution of injury following cerebral ischemia) [83]. MiR-181c was found to directly target tumor necrosis factor-alpha (TNF-α) after ischemia, thereby regulating microglia activation and microglia-mediated neuronal injury *in vitro* [84]. High expression of miR-181c was also shown to suppress expression of iNOS, leading to decreased production of nitric oxide (NO) following ischemic injury.

The miR-29 family consists of miR-29a, miR-29b, and miR-29c, distributed across the central nervous system and enriched in astrocytes [85]. All members have been shown to regulate various facets of inflammation [86]. For instance, inhibition of miR-29b significantly reduced the expression of activated microglial pro-inflammatory mediators such as TNF-α, interleukin 1b (IL-1b), interleukin 6 (IL-6), and monocyte chemoattractant protein-1 (MCP-1) [87]. Recently, miR-29b activity has been recognized as a survival factor for neurons after cerebral ischemia by suppressing the expression of the pro-apoptotic BH3-only proteins (BCL-2 homology domain 3) [88]. Interestingly, another study reported that an increase in miR-29b expression caused neuronal death in focal ischemia by inhibiting BCL-w, member of the Bcl-2 protein family [89]. Whether the same effect occurs in astrocytes or microglia is not known. However, for example, using TargetScan5.1 (http://www.targetscan.org/) miR-29b can be predicted to target several members of the Bcl-2 family known to have both protective and antagonistic properties. Thus, the multiple functions of miR-29b are likely related to which member of the Bcl-2 family exhibits a more dominant effect in a given cell type.

## 7 REGULATION OF MIRNAS IN RISK FACTORS

Risk factors for ICH, such as atherosclerosis and arterial hypertension, cause mitochondrial dysfunction, which leads to an overproduction of ROS and causes dysfunction of the endothelium, proliferation and apoptosis of VSMCs and macrophages, thereby contributing to the development of vascular hyperplasia with lipohyalinosis of the vascular wall, which in turn predisposes to rupture of the vessel [90-92]. Impaired mitochondrial structure and function result in a unique transcriptional response, in which dysregulated miRNAs may play a critical role in decreasing ATP production and enhancing ROS formation. In ICH process, mitochondrial ROS increased in ECs induced by a variety of factors, such as hypertension, hyperlipidemia or hyperglycemia [90-92]. There are many sources of ROS production in cells, and these include mitochondria, nicotinamide adenine dinucleotide phosphate (NADPH), or xanthine oxidase (XO), but it is mitochondria that are considered the main source of ROS in human EC [93]. ROS produced by mitochondria are the main promoter of cellular signals during stress stimulation or exposure to risk factors for ICH [94]. Many aspects of mitochondrial dysfunction in hypertension and atherosclerosis at the molecular level are still unknown. Elucidating these processes and identifying changes in the expression of certain miRNAs involved in the pathogenesis of ICH risk factors is a very valuable and exciting strategy, which may ultimately lead to the development of new approaches to ICH prevention. In the following sub-chapters, we will review the current knowledge of the role of miRNAs in mitochondria function of hypertension and atherosclerosis.

### 7.1 Atherosclerosis

In mitochondrial dysfunction, excessive production of ROS and nitrogen contributes to inflammatory vascular reactions leading to the development of atherosclerotic lesions [91]. ROS and nitrogen play an important role in atherogenesis, they are involved in processes such as dysfunction and apoptosis of ECs, activation of MMPs, phenotypic switch of VSMCs, and their migration into the intima, expression of adhesion molecules, and oxidation of low-density lipoproteins (LDL) [91]. All these processes contribute to the progression of atherosclerotic lesions. There is evidence that miRNAs are involved in mitochondrial dysfunction in the development of atherosclerosis [95]. Several studies have demonstrated that hypoxia inducible factor 1 alpha (HIF1a) reduces cellular ROS production by switching energy production from oxidative phosphorylation to glycolysis in several ways [96, 97]. HIF-1a also activates the transcription of genes encoding glucose transporters and glycolytic enzymes, which increases the flow from glucose to lactate [98]. HIF-1a activates the apoptotic protein Bcl-2 nineteen-kilodalton interacting protein 3 (BNIP3), which induces mitochondria-selective autophagy under hypoxic conditions [99]. In addition, HIF-1α plays a key role in inflammatory macrophage activation under normoxic conditions [100]. It is known to upregulate ROS production, the effect of HIF-1α on mitochondrial function is mediated by miRNAs. For instance, Karshovska *et al.* showed that HIF-1α mediates the switch of the mitochondrial function from ATP to ROS production in lipopolysaccharide (LPS)/interferon-γ (IFN-γ)-stimulated macrophages [101]. Furthermore, they found that HIF-1α-induced *via* miR-210 reduces mitochondrial respiration and increases ROS production and cell death in inflammatory macrophages. In addition, downregulation of miR-383 by HIF-1α in macrophages increased ATP depletion by suppressing poly (ADP-ribose) glycohydrolase, which enhanced atherosclerosis.

In another study, Wang *et al.* suggested that downregulation of miR-18a expression in oxidized low-density lipoprotein (ox-LDL) treated human aortic endothelial cells (HAECs) reversed mitochondrial energy metabolism disorder and upregulated peroxisome proliferator activated receptor (PPAR) γ-coactivator 1-α (PGC-1α), expression [102]. Moreover, it is known that PGC-1a is a key molecule that regulates mitochondrial function and mitochondrial energy metabolism [103]. In this study demonstrated that overexpression of PGC-1a can reverse the effect of ox-LDL and reduce the level of mitochondrial ROS and apoptosis. Moreover, downregulation of PGC-1a expression in normal HAECs could cause mitochondrial energy metabolism disorder, increase mitochondrial ROS level and apoptosis level. The most common miRNAs reported in the regulation of mitochondrial function in atherosclerosis are summarized in Table **2** [104-112].

### 7.2 Hypertension

Hypertension is a major risk factor for the development of ICH and it is associated with endothelial dysfunction and oxidative stress [90-92]. In addition, in hypertension, there is a disruption in the work of mitochondrial ATP synthase in ECs. Mitochondrial calcium overload makes a significant contribution to the development of hypertension [46]. There are only a few studies showing the regulatory role of miRNAs in mitochondrial function in hypertension. One of them is the study of Tan *et al.* In their study was demonstrated that miR-9 inhibited expression of MAM (mitochondria associated membrane) domain containing glycosylphosphatidylinositol anchor 2 (MDGA2) leading to the inhibition of apoptosis and promotion of proliferation in angiotensin II-treated human umbilical vein endothelial cells (HUVECs) [116]. It is known that MDGA2 is a transcription factor and being mitochondria-related gene, and its dysregulation related to several human diseases [117]. Also previously was demonstrated that overexpression of miR-9-5p contributed to the reduction of oxidative stress, cell apoptosis, and mitochondrial dysfunction by targeting GSK-3β in Alzheimer’s disease model [118]. The most common miRNAs reported in the regulation of mitochondrial function in hypertension are summarized in Table **2** [113-115].

## 8 THE NEED FOR BIOMARKERS IN ICH

The modern stroke diagnosis is based on clinical examination data and neuroimaging techniques [2]. Liquid biopsy looking for single biomarkers or multiple sets of them that can be used to diagnose acute stroke, to differentiate types of stroke (*e.g.*, non-traumatic hemorrhage from intracerebral hemorrhage due to ruptured intracranial aneurysms), or even to predict initial or recurrent stroke, may be very valuable. Modern diagnosis of ICH is difficult, diagnosis appears to be delayed due to the lack of a suitable mechanism for a rapid (ideally at the patient's bedside), accurate, and analytically sensitive diagnostic method based on the determination of biomarkers. There is a clear need for further research in this area. Potential biomarkers require rapid clinical confirmation of their use for early diagnosis, real-time diagnosis and prognosis ICH, which should improve the outcome of the disease and the quality of life of patients. One of the difficulties in the discovery of new biomarkers is associated with the solution of issues with overcoming the BBB. There are a number of studies that have demonstrated that the BBB can be an obstacle preventing proteins from entering the bloodstream [119]. In addition, current ICH biomarkers (*e.g.*, D-dimer) may lack diagnostic specificity and be overexpressed in other diseases [120]. Moreover, one of these directions in the search for new biomarkers in ICH is the study of circulating miRNAs.

### 8.1 Circulating miRNAs as Biomarkers

**Fig. (2):**
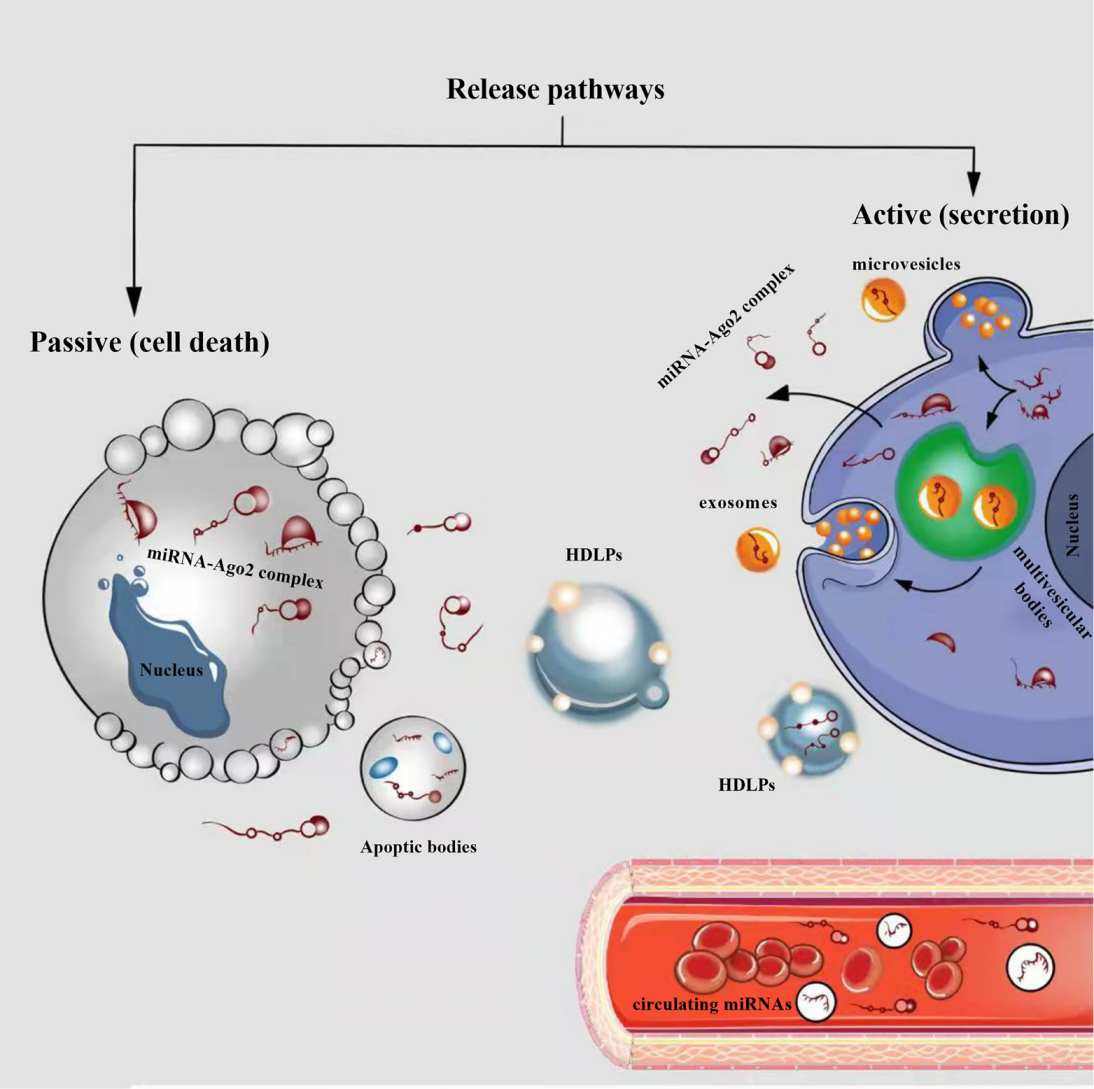
Biogenesis and function miRNAs and mechanisms of mitochondrial miRNAs (mitomiRs) transport and localization to mitochondria. MitomiRs are miRNAs of nuclear origin that are associated with the mitochondrial membrane and, possibly, with their own mRNA targets (**1**) or localized within organelles (**2**), like mit-mitomiRs (mitochondrial origins). MitomiRs suppress or activate gene expression by interacting with complementary sequences of their mRNA-targets, which are nuclear (**1**) or mitochondrial transcripts (**2**). Argonaute 2 protein (Ago-2) transports mitomiRs to mitochondria. Have been put forward about three main mechanisms: (**3**) exchange through contact between mitochondria and P-bodies; (**4**) transport *via* PNAPASE, component; or transport across the porins of the outer mitochondrial membrane.

Today, there are three known pathways for the release of miRNAs into biological fluids: (1) passive release from damaged cells, due to apoptosis or necrosis; (2) active secretion by extracellular vesicles (EVs), including exosomes and microvesicles (MVs); (3) active secretion or passive release using an RNA-binding protein-dependent pathway like the miRNA-Ago2 complex (Fig. **2**) [9, 121]. The expression profile of circulating miRNAs significantly changes (aberration or deregulation) in various human cardiovascular diseases, including ICH [122-125]. Several properties of circulating miRNAs support their potential use as non-invasive biomarkers in the field of the cardiovascular system, including high stability in biological fluids, sensitivity and specificity of the disease. More detailed information on circulating miRNAs is presented in our previous studies [126-129].

Since there are many mitochondria in the cells of the CNS, including the ECs of the BBB (the number of mitochondria in the ECs of cerebral vessels is 5-10 times higher than in the endothelium of peripheral vessels), the signature of mitochondrial miRNAs may reflect pathological states of mitochondrial metabolism and structures associated with the development of ICH [130, 131]. Thus, aberrant expression of mitochondrial miRNAs in biological fluids associated with altered mitochondrial function should be considered one of the main signs of ICH development. Due to the fact that a universal measurement method that allows easily, quickly, reliably, and inexpensively to determine mitochondrial miRNAs has not yet been developed, the introduction of circulating mitochondrial miRNAs as non-invasive biomarkers in clinical practice is still hypothetical. The identification and verification of changes in the expression of circulating miRNAs, unique for specific phases of the disease, their relationship with etiology, as well as the integration of multi-center clinical trials, can provide more clarity on the value of use as biomarkers. In addition, the possible combination of several circulating mitochondrial miRNAs can be considered as a starting point for further research.

Among the registered miRNAs, miR-21 is considered one of the important miRNAs, and it has been shown that there is a change in its expression in cardiovascular diseases, including strokes, which suggests that circulating miR-21 may be a potential biomarker [132]. For instance, the some studies identified changes in circulating miR-21 expression (*i.e.* downregulation) in both peripheral blood and haematoma samples from patients and animals with ICH [132-134]. Previous studies have reported that phosphatase and tensin homolog deleted on chromosome ten (PTEN) is activated in ECs after high glucose treatment, implying that PTEN may be a potential therapeutic target for high glucose-induced endothelial injury [135]. Zhang *et al.* demonstrated that the expression of miR-21 is associated with the inhibition of high glucose-mediated PTEN signaling in HUVECs, which decreased the NO production and COX-2 activation [136]. At the same time, it has been proven that hyperglycemia during hospitalization has a detrimental effect on the survival and functional outcome of ICH patients. In addition, Sala *et al.* demonstrated the association of miR-21 with oscillating and high glucose and early mitochondrial dysfunction in HUVECs [137]. The authors found that miR-21 may promote the suppression of homeostatic signalling (Krev1 interaction trapped gene 1 (KRIT1), forkhead box protein O1 (FoxO1), nuclear factor erythroid 2-related factor 2 (NRF2) and superoxide dismutase 2, mitochondrial (SOD2)) that normally limits ROS damage. Nevertheless, more studies are needed on the possible role of miR-21 in ICH, as well as a role in the regulation of mitochondrial dysfunction, and for the possible use of mitochondrial circulating miR-21 as a non-invasive biomarker.

## CURRENT AND FUTURE PERSPECTIVES

Although our knowledge of miRNAs in mitochondrial dysfunction is still incomplete, they appear to be key players in the regulation of nuclear and mitochondrial gene expression. Mitochondrial dysfunction caused by impaired mitochondrial-nuclear communication may play a role in the development of various cardiovascular diseases, including ICH. Understanding the functions of mitochondria under normal and pathological conditions is critical not only for understanding the causes of ICH but also for developing therapeutic strategies. If mitochondrial disorders directly or indirectly contribute to the development of pathological conditions, then the elimination of mitochondrial dysfunction should reduce the severity or slow down the progression of the disease. Further studies in this direction are presented necessary. Understanding the exact mechanisms by which miRNAs are involved in mitochondrial dysfunction and, as a result, mitochondrial dysfunction contributes to the development of damage to the cerebral vascular endothelium, will open up new methods of prevention and treatment.

The currently used technologies for diagnosing and predicting stroke, as well as the spectrum of biomarkers of vascular risk and endothelial damage, are not always sufficient to initiate timely treatment and choose the correct tactics. Known key role mitochondrial dysfunction of target cells of the cerebral vascular endothelium during prolonged exposure to high blood pressure and atherosclerotic lesions, as well as in the development of damage to the nervous tissue after an ICH. This predetermines the possibility of participation of structural and functional disorders of mitochondria of ECs, VSMCs, and monocytes/macrophages in various stages of the development of atherosclerosis and arterial hypertension. Despite this fact, biomarkers linking the state of mitochondria with the development of an ICH, to date have not been identified.

## CONCLUSION

One of the promising indicators of the intensity of cytolytic processes and, possibly, the state of mitochondria, are circulating miRNAs, including mitochondrial ones. Today, the detection of circulating miRNAs in biological fluids is being studied as new promising diagnostic and prognostic tools for malignant tumors, infectious diseases, cardiovascular diseases, as well as for assessing the likelihood of death in patients in intensive care units (ICU). At the same time, studies of the dynamics of changes in expression circulating mitochondrial miRNAs in ICH and its risk factors, such as arterial hypertension and atherosclerosis, have not been previously conducted. Thus, it is relevant for clinical neurology and neurosurgery to study the expression level of circulating miRNAs as a damage-associated molecular pattern in ICH. All this will create the basis for the creation of technologies for diagnostic and prognostic monitoring of acute vascular brain injuries. In conclusion, the above findings in the results of these studies might provide a novel experimental and theoretical basis for miRNAs regulation in mitochondrial dysfunction of ICH.

## Figures and Tables

**Table 1:** Some miRNAs with targets important to mitochondrial function in cardiovascular disease.

**miRNAs**	**Disease**	**Model**	**Targeting Mitochondria**	**Platform**	**Mirna Location**	**Expression**	**References**
miR-696, miR-532, miR-690, and miR-345-3p	HF	*In vivo*	Fatty acid biosynthesis, energy metabolism, and oxidative stress pathways	Bioinformatics analysis, RNA-seq and qRT-PCR	Mitochondria	Up	[24]
miR-195	HF	Human cells and *in vivo*	Energy metabolism and oxidative stress pathways (DLST)	Bioinformatics analysis and qRT-PCR	Mitochondria	Up	[25]
miR-181c	HF	*In vivo*	Energy metabolism and oxidative stress pathways (mt-COX1)	qRT-PCR	Mitochondria	Up	[26]
miR-29a	IA	Human whole blood, *in vivo* and *in vitro*	Mitochondrial apoptosis signaling (caspase-3, -8 and -9, and proteins, including cytochrome c and Mcl-1)	Bioinformatics analysis, microarray, and qRT-PCR	Cytosol	Up	[27]
miR-194	AAA	*In vivo* and *in vitro*	Oxidative stress pathways (KDM3A) and BNIP3)	Bioinformatics analysis and qRT-PCR	Cytosol	Down	[28]
miR-668	IS	*In vivo*	Mitochondrial apoptosis signaling pathway (Caspase 3, Bax, and Bcl-2) and oxidative stress pathways (NLRP3, ZO-1, and occludin proteins)	qRT-PCR	Cytosol	Down	[29]
miR-338	IS	*In vitro*	COX4I1	qRT-PCR	Cytosol	Up	[30]
miR-92b-3p	IS	*In vitro*	Repressed mitochondrial membrane potential depolarization, reactive oxygen species production, and cytochrome c protein expression (TRAF3)	qRT-PCR	Cytosol	Down	[31]

**Abbreviations:** HF, hear failure; IA, intracranial aneurysm; AAA, abdominal aortic aneurysm; IS, ischemic stroke; DLST, dihydrolipoamide succinyltransferase; mt-COX1, cytochrome c oxidase subunit 1; Mcl-1, myeloid cell leukemia 1; KDM3A, lysine demethylase 3A; BCL2, B-cell lymphoma 2; BNIP3, Bcl-2 interacting protein 3; Bax, Bcl-2-associated X protein; NLRP3, NOD-, LRR- and pyrin domain-containing protein 3; ZO-1, zonnula occludens 1; COX4I1, cytochrome-c oxidase subunit 4I1; TRAF3, tumor-necrosis factor receptor-associated factor 3; qRT-PCR, real-time reverse transcriptase polymerase-chain reaction; RNA-seq, RNA sequencing.

**Table 2:** Summary of recent studies on the role of miRNAs with targets in mitochondrial function in hypertension and atherosclerosis.

**miRNAs**	**Related Disease**	**Study Model**	**Type of Sample**	**Targets**	**Proposed Role**	**Expression**	**References**
miR-144	Atherosclerosis	*In vivo* and *in vitro*	HAECs and mice ECs	IDH2	Increase oxidant stress and decrease NO bioavailability.	Up	[104]
miR-210	Atherosclerosis	*In vitro*	HEK293 cell line	MEF2C	Inhibits hypoxia-induced apoptosis of VSMCs	Down	[105]
miR-33	Atherosclerosis	*In vitro*, human and *in vivo*	Human atherosclerotic plaque tissue and mouse macrophages	ABCA1, PGC-1α, SLC25A25, NRF1 and TFAM	Reduce cholesterol efflux *via* repression of mitochondrial energy metabolism pathways. Enhances mitochondrial respiration and ATP production	Up	[106]
miR-19b	Atherosclerosis	*In vitro*, human and *in vivo*	HAECs, human blood samples, mice ECs	PAI-1, STAT3 and SIRT3	Inhibit oxidant-induced endothelial dysfunction	Down	[107]
miR-19b-3p, miR-221-3p and miR-222-3p	Atherosclerosis	Human and *in vitro*	Human atherosclerotic and normal vessel samples, HUVECs and HAECs	PGC-1α	Modulate ECs apoptosis *via* the regulation mitochondrial function	Up	[108]
miR-26a	Atherosclerosis	*In vitro* and *in vivo*	HAECs and mice ECs	TRPC6	Inhibits ECs apoptosis *via* the regulation mitochondrial function	Down	[109]
miR-217-5p	Atherosclerosis	*In vitro* and *in vivo*	HAECs and mice ECs	CLIC4	Inhibits ECs apoptosis *via* the regulation mitochondrial function	Down	[110]
miR‐125a‐5p	Atherosclerosis	*In vitro*	HUVECs	TET2	Modulate abnormal DNA methylation, mitochondrial dysfunction, and increased reactive oxygen species production, and activated nuclear factor‐κB	Up	[111]
miR-34a	Atherosclerosis	Human and *in vitro*	Human serum and plaque tissue collection, and HUVECs	Bcl-2	Modulate ECs apoptosis *via* the regulation mitochondrial function and oxidative stress	Up	[112]
miR-18a- 5p	Hypertension	*In vivo*	Brain and cardiac tissue	HIF-1a	Mitochondrial biogenesis: increase mitochondrial stress proteotoxicity, decrease UPR^mt^ leading to decrease mitochondrial dynamics/OXPHOS/ mitochondrial membrane potential and ROS generation	Down	[113]
miR-106a	Hypertension	*In vivo* and *in vitro*	Cardiac tissue and HEK293 cell line	Mfn2	Induce mitochondrial membrane depolarization, ROS production, and mitochondrial cristae derangement	Up	[114]
miR-21	Hypertension	*In vivo*	Cardiac tissue	Cytb	Regulate mitochondrial translation	Up	[115]

**Abbreviations:** HEK293, human embryonic kidney 293; HAECs, human aortic endothelial cells; ECs, endothelial cells; HUVECs, human umbilical vein endothelial cells; HIF-1a, hypoxia-inducible factor-1α; UPR^mt^, mitochondrial unfolded protein response; Mfn2, mitofusin 2; IDH2, isocitratedehydrogenase2; PGC-1α, peroxisome proliferator-activated receptor γ coactivator 1α; Bcl-2, B-cell lymphoma 2; Cytb, cytochrome b; ABCA1, ATP binding cassette subfamily A member 1; PGC-1α, peroxisome proliferator-activated receptor gamma coactivator 1-alpha; SLC25A25, solute carrier family 25 member 25; NRF1, nuclear respiratory factor 1; TFAM, human mitochondrial transcription factor A; PAI-1, plasminogen activator inhibitor-1; STAT3, signal transducer and activator of transcription 3; SIRT3, sirtuin-3; PGC-1α, peroxisome proliferator-activated receptor-gamma coactivator-1alpha; TRPC6, transient receptor potential cation channel subfamily C member 6; CLIC4, chloride intracellular channel 4; TET2, tet methylcytosine dioxygenase 2; OXPHOS, oxidative phosphorylation; ROS, reactive oxygen species; VSMCs, vascular smooth muscle cells; NO, nitric oxide; ATP, adenosine triphosphate.

## LIST OF ABBREVIATIONS

ADP-ribose: ATP Depletion by Suppressing Poly Glycohydrolase
AGO: Argonaute
Ago2: Argonaute 2
ANT: Adenine Nucleotide Transporter
ATP: Adenosine Triphosphate
BBB: Blood-brain Barrier
Bcl-2: B-cell Lymphoma 2
BH3-only proteins: BCL-2 Homology Domain 3
BNIP3: Bcl-2 Nineteen-kilodalton Interacting Protein 3
Ca^2+^: Calcium Ions
CECs: Cerebrovascular Endothelial Cells
COX-2: Cyclooxygenase-2
CYBRD1: Cytochrome B Reductase 1
CYBRD5: Cytochrome B5 Reductase 3
CYCS: Cytochrome c
CyPD: Cyclophilin D
cytoCa^2+^: Cytosolic Ca^2+^ Signals
ECM: Extracellular Matrix
ECs: Endothelial Cells
EGFR: Epidermal Growth Factor Receptor
EPC-EXs: Endothelial Progenitor Cells
EVs: Extracellular Vesicles
FoxO1: Forkhead Box Protein O1
GV: Glycemic Variability
HAECs: Human Aortic Endothelial Cells
HDLP: High-density Lipoprotein Particles
HIF1a: Hypoxia Inducible Factor 1 Alpha
HUVECs: Human Umbilical Vein Endothelial Cells
ICH: Intracerebral Hemorrhage
ICU: Intensive Care Units
IFN-γ: Interferon-γ
IL-1b: Interleukin 1b
IL-6: Interleukin 6
IS: Ischemic Stroke
KRIT1: Krev1 Interaction Trapped Gene 1
LDL: Oxidation of Low-density Lipoproteins
LPS: Lipopolysaccharide
MBs: Microvesicles
mCa^2+^: Mitochondrial Ca^2+^
MCAO: Middle Cerebral Artery Occlusion
MCP-1: Monocyte Chemoattractant Protein-1
MCU: Mitochondrial Calcium Uniporter
MDGA2: MAM (Mitochondria Associated Membrane) Domain Containing Glycosylphosphatidylinositol Anchor 2
mDNA: Mitochondrial DNA
miRNAs: MicroRNAs
MPP+: 1-methyl-4-phenylpyridinium
mPTP: Mitochondrial Permeability Transition Pore
mRNA: Messenger RNA
NADH: Nicotinamide Adenine Dinucleotide
NADPH: Nicotinamide Adenine Dinucleotide Phosphate
NCX1: Sodium-calcium Exchanger-1
NO: Nitric Oxide
NRF2: Nuclear Factor Erythroid 2-related Factor 2
ox-LDL: Oxidized Low-density Lipoprotein
OXPHOS: Oxidative Phosphorylation
oxyHb: Oxyhemoglobin
PD: Parkinson Disease
PDK1 and PDK2: Pyruvate Dehydrogenase Kinase Isozyme 1 and 2
PGC-1α: γ-coactivator 1-α
PGE2: Prostaglandin E2
PI3K: Phosphoinositide 3-kinase
PPAR: Peroxisome Proliferator Activated Receptor
pre-miRNA: Precursor miRNA
pri-miRNA: Primary miRNA
PTEN: Tensin Homolog Deleted on Chromosome Ten
ROS: Reactive Oxygen Species
SDHC: Succinate Dehydrogenase Subunit c
SOD2: Superoxide Dismutase 2
SPG7: Spastic Paraplegia Type 7
STAT3: Signal Transducer and Activator of Transcription 3
TBI: Traumatic Brain Injury
TLR4: Toll-like Receptor 4
TNF-α: Tumor Necrosis Factor-alpha
TNRC6A: Trinucleotide Repeat-containing gene 6A protein
3'-UTR: 3'-Untranslated Region
VDAC1: Voltage-dependent Anion Channel 1
VSMCs: Vascular Smooth Muscle Cells
XIAP: X-linked Inhibitor of Apoptosis Protein
XO: Xanthine Oxidase
XPO5: Exportin 5
ZO-1: Zonula Occludens 1
